# Genetic predisposition influences plasma lipids of participants on habitual diet, but not the response to reductions in dietary intake of saturated fatty acids

**DOI:** 10.1016/j.atherosclerosis.2010.12.039

**Published:** 2011-04

**Authors:** C.G. Walker, R.J.F. Loos, A.D. Olson, G.S. Frost, B.A. Griffin, J.A. Lovegrove, T.A.B. Sanders, S.A. Jebb

**Affiliations:** a MRC Human Nutrition Research, Elsie Widdowson Laboratory, Fulbourn Road, Cambridge CB1 9NL, UK; b MRC Epidemiology Unit, Institute of Medical Science, Cambridge, UK; c Nutrition and Dietetic Research Group, Imperial College London, London, UK; d Faculty of Health and Medical Sciences, University of Surrey, Guildford, UK; e Hugh Sinclair Unit of Human Nutrition, University of Reading, Reading, UK; f Nutritional Sciences Division, Kings College, University of London, London, UK

**Keywords:** Dietary saturated fat, Plasma lipids, SNP, Genetic predisposition score, Lipoprotein, Gene–diet-interaction

## Abstract

**Objective:**

SNPs identified from genome-wide association studies associate with lipid risk markers of cardiovascular disease. This study investigated whether these SNPs altered the plasma lipid response to diet in the ‘RISCK’ study cohort.

**Methods:**

Participants (*n* = 490) from a dietary intervention to lower saturated fat by replacement with carbohydrate or monounsaturated fat, were genotyped for 39 lipid-associated SNPs. The association of each individual SNP, and of the SNPs combined (using genetic predisposition scores), with plasma lipid concentrations was assessed at baseline, and on change in response to 24 weeks on diets.

**Results:**

The associations between SNPs and lipid concentrations were directionally consistent with previous findings. The genetic predisposition scores were associated with higher baseline concentrations of plasma total (*P* = 0.02) and LDL (*P* = 0.002) cholesterol, triglycerides (*P* = 0.001) and apolipoprotein B (*P* = 0.004), and with lower baseline concentrations of HDL cholesterol (*P* < 0.001) and apolipoprotein A-I (*P* < 0.001). None of the SNPs showed significant association with the reduction of plasma lipids in response to the dietary interventions and there was no evidence of diet-gene interactions.

**Conclusion:**

Results from this exploratory study have shown that increased genetic predisposition was associated with an unfavourable plasma lipid profile at baseline, but did not influence the improvement in lipid profiles by the low-saturated-fat diets.

## 1 Introduction

Plasma lipids are risk factors for cardiovascular disease (CVD) and are known to be sensitive to dietary change [1]. Plasma cholesterol can be altered by changes to the quality and quantity of fat in the diet; in particular total plasma cholesterol can be lowered by reductions in dietary total or saturated fat [2,3]. However, replacement of SFA with carbohydrate also reduces HDL cholesterol (HDL-C) with an associated increase in CVD risk [4]. Replacement of SFA with monounsaturated fatty acids (MUFA) achieves a decrease in total (TC) and LDL cholesterol (LDL-C) without a decrease in HDL-C [4,5]. Genetic factors also exert a strong influence on the regulation of plasma lipids, with heritability estimates for fasting plasma lipids ranging from 35 to 60% [6–8]. There is growing interest in the interplay between genetic and environmental factors, which may help to explain the variation between individuals in response to diet [6].

Genome-wide association (GWA) studies have identified a number of SNPs robustly associated with traits of dyslipidaemia in cross-sectional studies [9–16]. However, it remains unknown whether these common lipid-associated SNPs also alter the responses to dietary interventions. We hypothesised that the mechanisms underlying genetic predisposition to dyslipidaemia would impair the improvement in plasma lipid status which can be produced by modifying the amount and type of dietary fat. This hypothesis was tested in a cohort of 490 participants in the RISCK trial; a highly controlled intervention to reduce dietary saturated fat based on replacement with either carbohydrate (CHO) or monounsaturated fat (MUFA) [2]. We examined the association of 39 lipid-associated SNPs, individually as well as combined, on plasma lipid measures at baseline and on the change in response to 24 weeks dietary intervention.

## 2 Methods

### 2.1 Original RISCK trial study design

Full details of the RISCK trial have been published elsewhere [2]. Briefly, men and women aged 30–70 years (*n* = 720) were recruited from the general population. To participate in the trial, subjects had to be at increased risk of developing metabolic syndrome and CVD according to a study-specific scoring system [2]. Self-reported ethnicity was recorded as White; South and South East Asian, Black African, or other.

The reference and intervention diets (described in detail in Moore et al. [17]) were designed to be iso-energetic, but varied in the amount and type of fat and carbohydrate. For the purposes of the current study the dietary intervention groups differing in carbohydrate quality were combined to focus the analyses on the manipulation of dietary fat, from which the impact in CVD risk was expected to be greater. The resulting three dietary groups were; ‘reference diet’ (REF) designed to reflect saturated fat intake in a ‘Western diet’ (∼18% of energy (E) SFA, 12% MUFA, 38% total fat, 45% CHO); ‘MUFA diet’ in which SFA was reduced and replaced with MUFA (∼10% SFA, 20% MUFA, 38% total fat, 45% CHO); ‘LF diet’, in which SFA was reduced through replacement of total fat with carbohydrate (∼10% SFA, 11% MUFA, 28% total fat, 55% CHO).

All participants underwent a 4-week run-in period on the REF diet, after which anthropometry was measured and fasting blood samples were taken. Measurements taken after the run-in diet are referred to in this study as ‘baseline’ measurements. All participants followed their randomly prescribed diets for 24 weeks, after which a further blood sample was collected and anthropometry measured.

Ethical approval for the RISCK study (ISRCTN29111298) was granted from the National Research Ethics Service and written informed consent from participants was obtained including subsequent genetic analyses.

### 2.2 Characteristics of study cohort

Of the 720 participants, 549 completed the study and DNA was available for 512 participants. Based on self-reported ethnicity, we distinguished individuals of White (80%), South and South East (S, SE) Asian (9.5%); Black African (8%) and “other” (2.5%) ancestry. Analyses were stratified by self-reported ethnicity into three sub-groups with participants in the “other” subgroup excluded from this analysis (*n* = 11). The baseline characteristics of the participants in this study, stratified by the three main ethnic groups, are presented in Table 1.

The majority (91%) of the participants were overweight or obese and/or had elevated waist circumference (>94 cm for males and >84 cm for females) indicating the presence of central obesity.

We observed no significant differences for age, gender, BMI, body fat (%), waist circumference, glucose, triglycerides, apo B or apo A-I between the ethnic groups. Insulin concentrations were higher in S, SE-Asians compared to Whites, TC higher in Whites compared to S, SE-Asians and Black Africans and LDL-C and HDL-C lower in S, SE-Asians compared to Whites (Table 1).

### 2.3 Plasma lipid analyses and response to dietary intervention

TC, HDL-C and triglyceride (TG) were analysed at King's College London by enzymatic assay on a Bayer Advia Model Analyser using reagents supplied by the manufacturer (Bayer Diagnostics Europe, Newberry, Berks, UK). LDL-C was calculated using the Friedwald formula only it fasting TG concentrations were <4.49 mmol/L. Plasma apolipoproteins B (apo B) and A-I (apo A-I) were determined by immunoprecipitation assays (Randox Laboratories, Crumlin, UK) at the University of Surrey (see Jebb et al. [2] for further details).

As reported in full in Jebb et al. [2] plasma TC, LDL-C and apo B were significantly reduced in response to 24 weeks on the LF and MUFA diets. There was also a significant reduction in plasma HDL-C and apo A-I in response to the LF but not the MUFA diet. There was no change in plasma TG.

### 2.4 SNP selection and genotyping

Forty lipid-associated SNPs were identified from GWA studies published prior to April 2009. SNPs were only selected from GWA studies with at least 1000 individuals in the discovery stage, with replication in at least one independent population, and which reached the threshold of genome-wide significance of *P* < 5 × 10^−8^ [9–16]. Priority was given to SNPs that were plausible biological targets of lipid metabolic pathways, and that were identified by at least two independent GWA studies or meta-analyses. Where multiple SNPs resided in or near the same gene, SNPs in low linkage disequilibrium (LD *r*^2^ < 0.3) were selected with a maximum of three SNPs per gene.

Genotyping was performed in the 501 participants of the three main ethnic groups who completed the study, and for which there was DNA available. Genotyping was performed by KBiosciences (Hoddesdon, Herts, UK) using a fluorescence-based competitive allele-specific PCR (KASPar) technology and all SNPs had a call rate >95%. Individuals were excluded if genotyping was unsuccessful in >10% of SNPs (11 subjects). All genotype distributions were tested for deviation from the Hardy–Weinburg equilibrium using the log likelihood ratio chi-square test for association (*P* < 0.001); and one SNP was excluded (rs174547) from analyses due to deviation (*P* < 0.0001). This resulted in 14 HDL-C-associated SNPs in or near 11 genes; 12 LDL-C-associated SNPs in or near 11 genes; 5 TC-associated SNPs in or near 5 genes; and 10 TG-associated SNPs in or near 9 genes (Supplementary Table 1). Some SNPs were associated with more than one lipid trait.

### 2.5 Genetic predisposition score

A risk-allele was defined as the allele associated with raised TC, LDL-C, TG or low HDL-C in previous GWA studies [9–16]. An additive model was assumed and individual SNPs were coded as 0, 1 and 2 on the basis of the number of the risk-alleles for that particular SNP based on previous GWA studies [9–16]. As there is currently no evidence for interaction between SNPs, a simple addition of the associated risk alleles for each trait has been commonly adopted [18–20]. For each individual, genetic predisposition scores (GPS) were calculated for each of the traits separately (HDL-C, LDL-C, TC, TG) by adding all of the scores for each risk-allele associated with that trait (Supplementary Table 1). We did not weight the risk alleles on the basis of their individual effect sizes because it has been shown that weighting of risk alleles may have only limited effect [21] and because the effect size of SNPs on traits at baseline may not be relevant to the effect on the change in trait. We therefore elected for the approach employed previously [19] of simple addition of risk alleles allowing for a more straightforward interpretation. For participants missing individual genotyping data, the average count of risk alleles for the respective SNP was substituted for the missing genotype for the purposes of calculating the GPS. All GPS were normally distributed.

The SNPs selected for analysis are presented in Supplementary Table 1 showing the risk-allele frequency in each ethnicity for this cohort. Previous studies have shown that LDL-C-associated SNPs also associate with apo B and HDL-C-associated SNPs associate with apo A-I [16,18]. Therefore LDL-C-associated SNPs and LDL-C-GPS were also used to examine the effect on apo B and HDL-C-associated SNPs and HDL-C-GPS were used to assess the association with apo A-I.

### 2.6 Statistical analysis

Distributions of traits were tested for normality; and baseline TG was log-transformed for analyses and presented in tables in this form except for the subject characteristics in Table 1 where the geometric mean of TG was shown.

Linear regression analysis was used to test for associations between each SNP (coded as 0, 1 and 2 according to the number of risk-alleles) and the relevant traits at baseline, assuming an additive effect of each additional risk allele, while adjusting for age, gender and BMI. Due to the size of our cohort, we did not have sufficient power to detect significant associations between individual SNPs and lipid traits at baseline (Supplementary Fig. 1). For example SNP rs2338104, which has a minor allele frequency (MAF) of 47% in the cohort of white subjects has >80% power to detect an increase in trait of 0.2 SD-score per allele, but only 5% power to detect an increase in trait of 0.01 SD-score per allele. SNP rs1800961, which has a MAF of 3% in the cohort of white subjects has <25% power to detect an increase in trait of 0.2 SD-score per allele and <5% to detect an increase in trait of 0.01 SD-score per allele.

Next, we tested for association between each SNP and change in lipid concentration following 24 weeks of intervention (change calculated as final value minus baseline), adjusted for baseline values of respective trait, age, gender and BMI. There was no linearity assumption of relevant traits with diet, therefore the LF and MUFA diets were added in the model individually, in comparison to the REF diet.

Due to insufficient power to examine associations of individual SNPs, we focussed our study on the GPS, which provides more power. Associations between the GPSs and respective traits (baseline and change) were tested with linear regression in the same way individual SNPs were tested, adjusting for the same covariates.

All baseline and change associations were tested for interactions between gender and GPS and gender and individual SNP. Associations with change in traits were also tested for interactions between diets and GPS and diets and individual SNP.

All analyses were stratified by the three main ethnic groups. In order to obtain an overall result for the cohort, summary statistics of the ethnicity-specific associations were pooled using a random-effects meta-analysis based on the method of DerSimonian and Laird [22] (metan function in Stata). In order to determine whether the results of the three ethnicities could be combined, a test for heterogeneity was performed and the results of the meta-analysis were not presented if the associations between GPS or SNP and lipid trait were different between the ethnicities (*P* < 0.05 for the test of heterogeneity).

Statistical analysis was conducted using Stata 11 (StataCorp, Texas, USA). We performed a large number of tests but elected to not use any correction for multiple testing as this was an exploratory study to examine genetic predisposition to dyslipidaemia, and the interaction between GPS and lipids in response to a dietary intervention.

## 3 Results

### 3.1 Effect of genetic predisposition on lipids and apolipoproteins at baseline

The trait-specific GPSs were all associated with the respective traits, i.e. the higher the score the less favourable the lipid profile (Table 2). Each additional risk-allele in the TC-GPS was associated with 0.08 mM higher TC concentration; the LDL-C-GPS was associated with a 0.06 mM higher LDL-C concentration per additional risk-allele and the TG-GPS with a 0.04 mM higher lnTG concentration per additional risk-allele (Table 2).

**Fig. 1:**
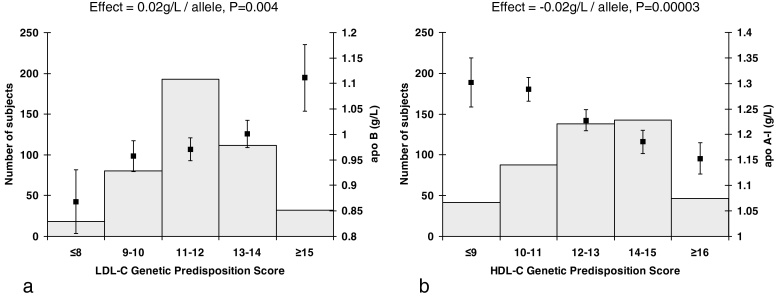
(a) Variation of apo B at baseline by LDL-C genetic predisposition score (GPS) and (b) variation of apo A-I at baseline by HDL-C genetic predisposition score (GPS). The squares represent the mean and standard error values of (a) apo B and (b) apo A-I (right *y*-axes) for each GPS score category defined by the number of (a) LDL-C and (b) HDL-C risk alleles per individual (*x*-axes). The histograms denote the number of individuals in each GPS score category (left *y*-axes).

The LDL-C-GPS was also associated with higher apo B concentration and the HDL-C-GPS with lower apo A-I levels per additional risk allele (Fig. 1).

We observed association coefficients which, although generally not significant, were directionally consistent with previous GWA studies for four out of five individual TC SNPs; for nine out of 12 LDL-C SNPs; for nine out of 13 HDL-C SNPs and for five out of seven TG SNPs, when data of all three ethnic groups were combined (Supplementary Table 2).

The association coefficients of 10 out of 12 LDL-C risk SNPs were in a positive direction with apo B, although not generally significant (Supplementary Fig. 2a). Eleven out of 13 HDL-C risk SNPs were in a negative direction with apo A-I (Supplementary Fig. 2b), four of which were also negatively associated with HDL-C (rs1800775 and rs9989419 – CETP locus; rs10468017 – LIPC locus; rs4846914 – GALNT2 locus).

There was little evidence of heterogeneity between the ethnic groups except two TG SNPs and one HDL-C SNP that was directionally consistent in Whites and S, SE Asians but not in Black Africans (Supplementary Table 2).

There were no significant interactions between gender and GPS or individual SNPs on the association with lipid traits at baseline (data not shown).

### 3.2 Effect of genetic predisposition on the change in lipids and apolipoproteins in response to dietary intervention to lower SFA

**Fig. 2:**
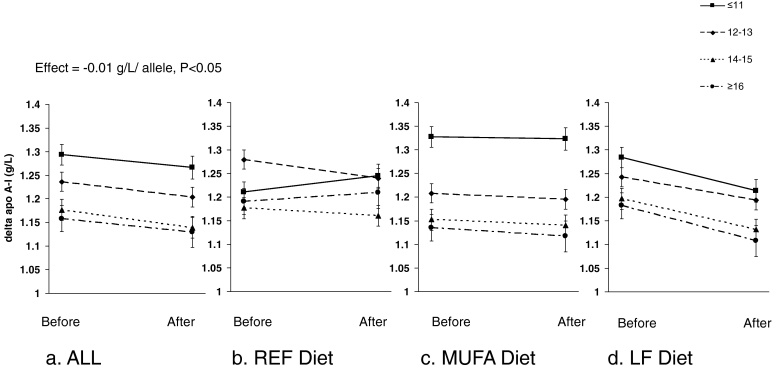
Apo A-I stratified by genetic predisposition score (HDL-C-GPS) before and after the dietary intervention in the total cohort, and split into dietary intervention groups. Data are presented as mean ± SE before and after 24 weeks on a dietary intervention for (a) the response in the total cohort and split into (b) the REF diet group; (c) the high MUFA diet group; and (d) the LF diet group. HDL-C-GPS was stratified into 4 groups [≤11; 12–13; 14–15; ≥16 risk alleles]. The number of subjects in each group was as follows: total (GPS-1:129, GPS-2:138, GPS-3:149, GPS-4:53); REF (GPS-1:17, GPS-2:28, GPS-3:22, GPS-4:9); MUFA (GPS-1:58, GPS-2:54, GPS-3:57, GPS-4:29); LF (GPS-1:54, GPS-2:56, GPS-3:70, GPS-4:15).

Following the dietary intervention, the decrease in apo A-I was greater the higher the HDL-C-GPS, with an effect size of −0.01 g/L per HDL-C risk allele (*P* < 0.05) (Table 3). The lipid-lowering effects of the LF diet and HDL-C-GPS were additive (Fig. 2) but there was no significant diet × HDL-C-GPS interaction effect for apo A-I (*P* > 0.09 for all ethnicities) or any other traits (data not shown). TC tended (*P* = 0.1) to be further reduced by 0.03 mM per allele with higher TC-GPS (Table 3). There was also a trend (*P* = 0.09) towards a greater reduction in HDL-C of 0.01 mM per allele with higher HDL-C-GPS. TG-GPS was not associated with change in TG or LDL-C-GPS with reduction of LDL-C or apo B (Table 3).

There was no evidence of heterogeneity across the three ethnic groups for the effect of any GPS on the change in lipid or apolipoprotein traits.

Of the five TC SNPs, one in the APOB locus tended (*P* = 0.06) to augment the diet-induced reductions in TC levels; of the 11 LDL-C SNPs, only one in the PCSK9 locus was associated (*P* = 0.04) with impeding the diet-induced reductions in LDL-C. No individual SNPs were associated with change in HDL-C, TG, apo B or apo A-I following the dietary intervention (Supplementary Table 3).

There was some indication (*P* < 0.1) of heterogeneity between the ethnic groups for the effect of individual SNPs on change in traits with one TC SNP on the change in TC; 1 LDL-C SNP on the change in LDL-C and four on the change in apo B; one HDL-C SNP on change in HDL-C and two on change in apo A-I (Supplementary Table 3). Generally, there was directional consistency between results in the Whites and S, SE Asians but not Black-Africans.

There was an interaction effect between gender and rs6511720 (*P* = 0.002) and the change in apo B, but no other significant interactions between gender and GPS or individual SNPs on the association with change in lipid traits.

While the intervention diets were designed to be iso-energetic, there was a small (<1 kg) but statistically significant (*P* < 0.001) incidental weight loss on the low fat diet during the trial [2]. As weight loss has been shown to improve lipid profiles [23], the analyses were repeated with and without weight change. There was no difference in the effect of any SNP or GPS on change in plasma lipids (data not shown).

## 4 Discussion

Our results show that genetic predisposition scores (which defined genetic susceptibility) were significantly associated with the respective lipid trait at baseline on a ‘reference’ diet high in SFA. We also confirmed that many of the SNPs identified in GWA studies showed effects of a similar magnitude and direction in this cohort of subjects at increased cardiometabolic risk, although typically not reaching significance in this study. We found no evidence that genetic predisposition to dyslipidaemic traits impaired the beneficial effects of a dietary intervention of reduced SFA intake to lower plasma TC, LDL-C and apo B.

This is the first study to examine how SNPs previously shown to be unequivocally associated with fasting lipid concentrations, modify lipid responses to dietary interventions. Due to the nature of highly controlled dietary interventions, we were limited to a relatively small sample size for investigating dietary-gene interactions. However, in terms of highly controlled dietary interventions this cohort was relatively large, providing the opportunity to undertake a preliminary investigation into the effect of these SNPs on dynamic responses to changes in dietary fat in a highly controlled situation.

Very few studies have examined the relationship between commonly occurring SNPs and plasma apolipoproteins. Previous GWA studies [16] and studies using the CardioChip [18] showed a close association between LDL-C-SNPs and apo B and HDL-C-SNPs and apo A-I. In the RISCK study cohort, we have shown that LDL-C-GPS (a cumulative GPS, composed of LDL-C-SNPs), was positively associated with apo B and that HDL-C-GPS (a cumulative GPS composed of HDL-C-SNPs) was negatively associated with apo A-I at baseline. In response to 24 weeks dietary intervention both the LF diet (*P* < 0.05) and the HDL-C-GPS (*P* < 0.05) were associated with a reduction in apo A-I. Although not significant, the association of HDL-C-GPS on change in HDL-C (*P* = 0.09) was also negative. A disadvantage of a low fat diet can be a resultant decrease in HDL-C [4], as low HDL-C and apo A-I are in themselves markers of metabolic syndrome and CVD risk [24–26]. This small preliminary study suggests that those genetically predisposed to low HDL-C, and who also have low apo A-I may have further adverse effects on lipid profile following a LF diet. However, as there was no adjustment for multiple testing in this study, these results are to be viewed as preliminary, and further research is warranted to explore the role of these common SNPs as ‘effect modifiers’ in dietary intervention studies to improve plasma lipids and apolipoproteins.

In this study, we focussed on SNPs with the strongest associations in GWA studies rather than selecting SNPs with known functionality. Furthermore, the opportunities to examine single SNPs were limited due to small sample size and the small effect size of the SNPs. We therefore focussed on the cumulative genetic predisposition using the GPS. Nonetheless, it is of note that many of the selected SNPs were located in or near genes involved in lipid metabolic pathways [6]. Many SNPs were also located in genes where rare monogenic mutations underlie causes of severe dyslipidaemia [27]. Notably, SNPs of CETP (Cholesteryl Ester Transfer Protein) and LIPC (hepatic lipase) loci were significantly associated with baseline HDL-C and apo A-I as found previously both in GWA studies [9,11,13,16] and in candidate gene studies [28,29]. However, these individual SNPs did not significantly alter the HDL-C and apo A-I response to dietary intervention. The rs4846914 SNP from the GALNT2 locus was also significantly associated with both baseline HDL-C and apo A-I in the current study. An association between this SNP and HDL-C was recently reported in a large meta-analysis of GWA studies of common variants and lipid traits, with follow-up functional studies in mice that implicated GALNT2 as a biological mediator of HDL-C concentration [30]. In the current study, there was also no association of this SNP with change in apo A-I, but it was associated with an increase (0.03 ± 0.01 mM, *P* = 0.03) in HDL-C in the White participants only, indicating a potential cardio-protective effect (Supplementary Table 3). It is noteworthy that many of these SNPs reside outside the coding regions of genes and thus the effects on lipid changes are difficult to interpret.

In the current study, there was little evidence of heterogeneity for individual SNPs or GPSs on lipid traits between different ethnic groups, which is consistent with a recent large study that showed no evidence of heterogeneity between European, South and South-East Asian and African American populations in 95 lipid-related SNPs [30]. There was a greater degree of heterogeneity between ethnic groups in the effects of SNPs on the change in lipid traits, however this finding can only be viewed as exploratory in view of the very small sample size of Asian and Black subgroups. Nevertheless preliminary results from this study may indicate that the effects of some SNPs in response to diets were greater in some ethnicities. For example SNPs rs693 (APOB locus) and rs3846662 (HMGCR locus) had a greater reduction in plasma TC (effect size −0.26 and −0.31 mM per risk allele) in Black-Africans group but no significant effect in Whites (Supplementary Table 3). As many of the SNPs reside outside coding regions of the genes, this may reflect that these SNPs were simply markers for other SNPs, and that the LD between the marker and functional SNP varies with ethnicity [31]. However, due to the very small sample size of the Black-African and South and South-East Asian subgroups in particular, any preliminary observations require investigation in larger cohorts.

The RISCK study was designed to represent achievable public health goals in terms of the manipulation of dietary fat. The current study indicates that regardless of common genetic predisposition to dyslipidaemia, improvements in lipid profile can be achieved by changes to dietary fat quality and content. This analysis on SNPs which may moderate lipid responses to diet is not exhaustive; however it covers SNPs most important for regulating lipids at a population level and may therefore have public health implications for the management of common dyslipidaemia by dietary means.

In conclusion, high plasma TC, TG, LDL-C and apo B and low apo A-I and HDL-C were influenced by genetic predisposition in this cohort of overweight men and women, who were identified at increased cardiometabolic risk. A dietary manipulation to lower SFA was successful in lowering plasma TC, LDL-C, and apo B, most notably in those genetically predisposed to dyslipidaemia.

## Source of funding

The RISCK dietary intervention trial was funded by the Food Standards Agency project number NO2031. Genetic analyses were funded by the participating centres.

## Figures and Tables

**Table 1:** Characteristics of subjects included in this study.

	White^a^ (*n* = 395)	S, SE Asian^a^ (*n* = 46)	Black African^a^ (*n* = 38)
Gender (M/F %)	59/41	64/36	59/41
Age (years)	53.0 (9.9)	50.5 (8.7)	50.5 (9.5)
BMI (kg/m^2^)	29.0 (4.8)	28.0 (4.2)	30.1 (5.4)
Body fat (%)	34.1 (8.3)	33.2 (8.6)	35.0 (8.2)
Waist circumference (cm)	98.0 (12.4)	97.1 (16.3)	100.7 (12.9)
Glucose (mM)	5.69 (0.77)	5.67 (0.83)	5.44 (0.84)
Insulin (pM)^b^	58.3 [55.4, 61.3]	72.0 [59.8, 86.8]*	68.3 [57.4, 81.3]
TC (mM)	5.74 (0.97)	5.19 (0.89)*	5.23 (1.00)*
HDL-C (mM)	1.41 (0.32)	1.28 (0.28)*	1.29 (0.29)
LDL-C (mM)	3.63 (0.81)	3.31 (0.79)*	3.32 (0.86)
TG (mM)^b^	1.41 [1.35, 1.47]	1.21 [1.07, 1.36]	1.25 [1.09, 1.43]
apo B (g/L)	0.99 (0.30)	0.96 (0.28)	0.91 (0.25)
apo AI (g/L)	1.23 (0.26)	1.22 (0.21)	1.23 (0.25)
TC:HDL-C (mM)	4.22 (0.92)	4.19 (0.92)	4.17 (0.97)

Data presented as mean (SD) unless stated otherwise. All variables were measured at baseline (following one month run-in on the REF diet). Differences between groups were tested by ANOVA with Tukey's post hoc test performed if *P* < 0.05. Differences between Whites and S, SE-Asians or Black-Africans were marked with * (*P* < 0.05). There were no significant differences between S, SE-Asians and Black-Africans.

a Stratified by self-reported ethnicity.

b TG and insulin data not normally distributed therefore presented as the geometric mean [95% confidence intervals].

**Table 2:** Effect of genetic predisposition score (GPS) on total, LDL and HDL cholesterol, triglycerides, apo A-I and apo B at baseline in three ethnicities and meta-analysed.

	White				S, SE-Asian				Black African				Meta-analysis				
	Effect	SE	*P*	*n*	Effect	SE	*P*	*n*	Effect	SE	*P*	*n*	Effect	95%CI	95%CI	*P*	Heterogeneity
TC (mM)	0.09	0.04	0.03	394	0.13	0.12	0.28	46	−0.04	0.13	0.74	39	0.08	0.01	0.15	0.02	0.56
LDL-C (mM)	0.06	0.02	0.009	392	0.09	0.05	0.09	46	0.07	0.09	0.46	39	0.06	0.02	0.10	0.002	0.80
HDL-C (mM)	−0.03	0.01	0.00002	394	−0.06	0.01	0.00005	46	−0.04	0.02	0.10	39	−0.03	−0.04	−0.02	8.9×10^−9^	0.10
lnTG (mM)	0.04	0.01	0.004	393	0.03	0.03	0.43	46	0.04	0.04	0.26	39	0.04	0.02	0.06	0.001	0.92
apo A-I (g/L)	−0.02	0.01	0.001	375	−0.04	0.01	0.005	46	−0.02	0.02	0.40	37	−0.02	−0.03	−0.01	0.00003	0.40
apo B (g/L)	0.02	0.01	0.005	375	0.02	0.02	0.23	46	0.01	0.02	0.68	37	0.02	0.01	0.03	0.004	0.93

Data are the co-efficient of associations of genetic predisposition score (GPS) for each trait, presented as per allele effect size, standard error and P derived from linear regression models. The linear regression models were of GPS against trait at baseline adjusted for age, gender and BMI. The LDL-C GPS was used for apo B and the HDL-C GPS for apo A-I regression analysis. TG data was logged for the analysis and is presented as log-transformed data. Data are presented for each ethnic sub-group. The summary statistics were meta-analysed and the per allele effect size (ES), 95% confidence intervals (CI), P and measure of heterogeneity (*P*-value from test of heterogeneity) are shown. The number of participants (*n*) in each subgroup is indicated.

**Table 3:** Effect of genetic predisposition score (GPS) on the change in total, LDL and HDL cholesterol, triglycerides, apo A-I and apo B following 24 weeks dietary intervention in three ethnicities and meta-analysed.

	White				S, SE-Asian				Black African				Meta-analysis				
	Effect	SE	*P*	*n*	Effect	SE	*P*	*n*	Effect	SE	*P*	*n*	Effect	95%CI	95%CI	*P*	Heterogeneity
ΔTC (mM)	−0.03	0.02	0.28	370	0.00	0.07	0.96	46	−0.14	0.07	0.047	38	−0.03	−0.075	0.007	0.10	0.25
ΔLDL-C (mM)	−0.01	0.01	0.38	368	0.01	0.03	0.67	46	0.00	0.06	0.94	38	−0.01	−0.030	0.015	0.51	0.76
ΔHDL-C (mM)	−0.01	0.00	0.24	370	−0.01	0.01	0.58	46	−0.02	0.01	0.18	38	−0.01	−0.012	0.001	0.09	0.66
ΔTG (mM)	0.01	0.01	0.66	369	−0.02	0.03	0.63	46	−0.03	0.05	0.55	38	0.00	−0.028	0.027	0.99	0.67
Δapo A-I (g/L)	−0.01	0.00	0.02	375	0.01	0.01	0.40	46	−0.01	0.02	0.78	37	−0.01	−0.013	0.000	0.048	0.28
Δapo B (g/L)	−0.01	0.00	0.27	375	0.00	0.01	0.94	46	0.02	0.02	0.28	37	0.00	−0.012	0.005	0.46	0.39

Data are the co-efficient of association of genetic predisposition score (GPS) for each trait with the change in that trait following 24 weeks dietary intervention, presented as per allele effect size, standard error and P derived from linear regression models. The linear regression models were of GPS against the change in trait adjusted for that trait at baseline, age, gender and BMI. The LDL-C GPS was used for apo B and the HDL-C GPS for apo A-I regression analysis. Data for change values were calculated by final value − baseline value, and negative effects represent an association with a reduction in trait following 24 weeks intervention. Data are presented for each ethnic sub-group. The summary statistics were meta-analysed and the effect size, 95% confidence intervals (CI), *P* and measure of heterogeneity (*P*-value from test of heterogeneity) are shown. The number of participants (*n*) in each subgroup is indicated.
