# Supplementary material for: Genetic predisposition influences plasma lipids of participants on habitual diet, but not the response to reductions in dietary intake of saturated fatty acids
Source: Atherosclerosis. 2011 Apr;215(2):421–7. doi: 10.1016/j.atherosclerosis.2010.12.039 (PMC3407860; doi:10.1016/j.atherosclerosis.2010.12.039)
Supplement: Supplementary file 1 [file mmc1.pdf]

# Supplementary figure 1

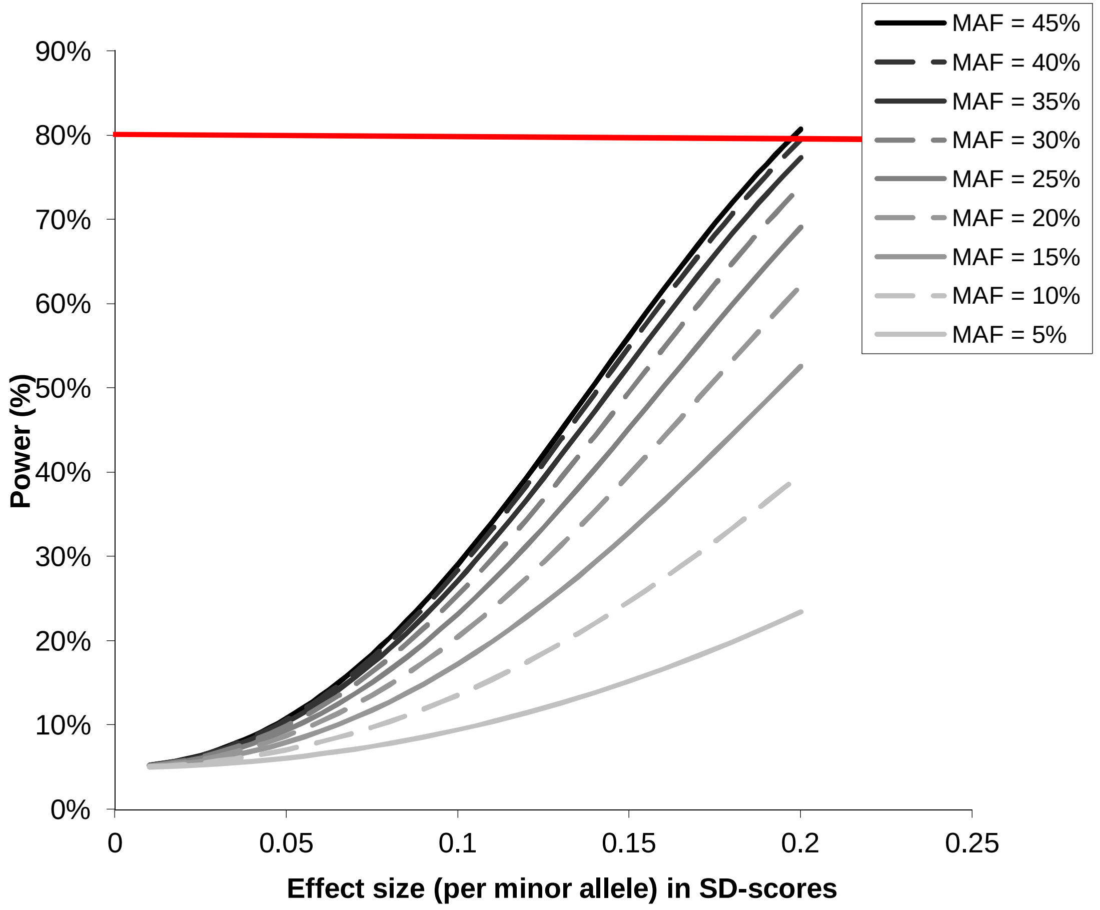


**Supplementary figure 1**

The power to detect various levels of effect sizes for a range of minor allele frequencies. A range of effect sizes for the difference in trait per allele are expressed as SD-scores and the corresponding power was calculated using Quanto (Version 1.2.4). This calculation was performed for allele frequencies from 5-45%. The calculation was based on the size (n~400) of the White population in this study.
