# Supplementary material for: Genetic predisposition influences plasma lipids of participants on habitual diet, but not the response to reductions in dietary intake of saturated fatty acids
Source: Atherosclerosis. 2011 Apr;215(2):421–7. doi: 10.1016/j.atherosclerosis.2010.12.039 (PMC3407860; doi:10.1016/j.atherosclerosis.2010.12.039)
Supplement: Supplementary file 2 [file mmc2.pdf]

Supplementary figure 2

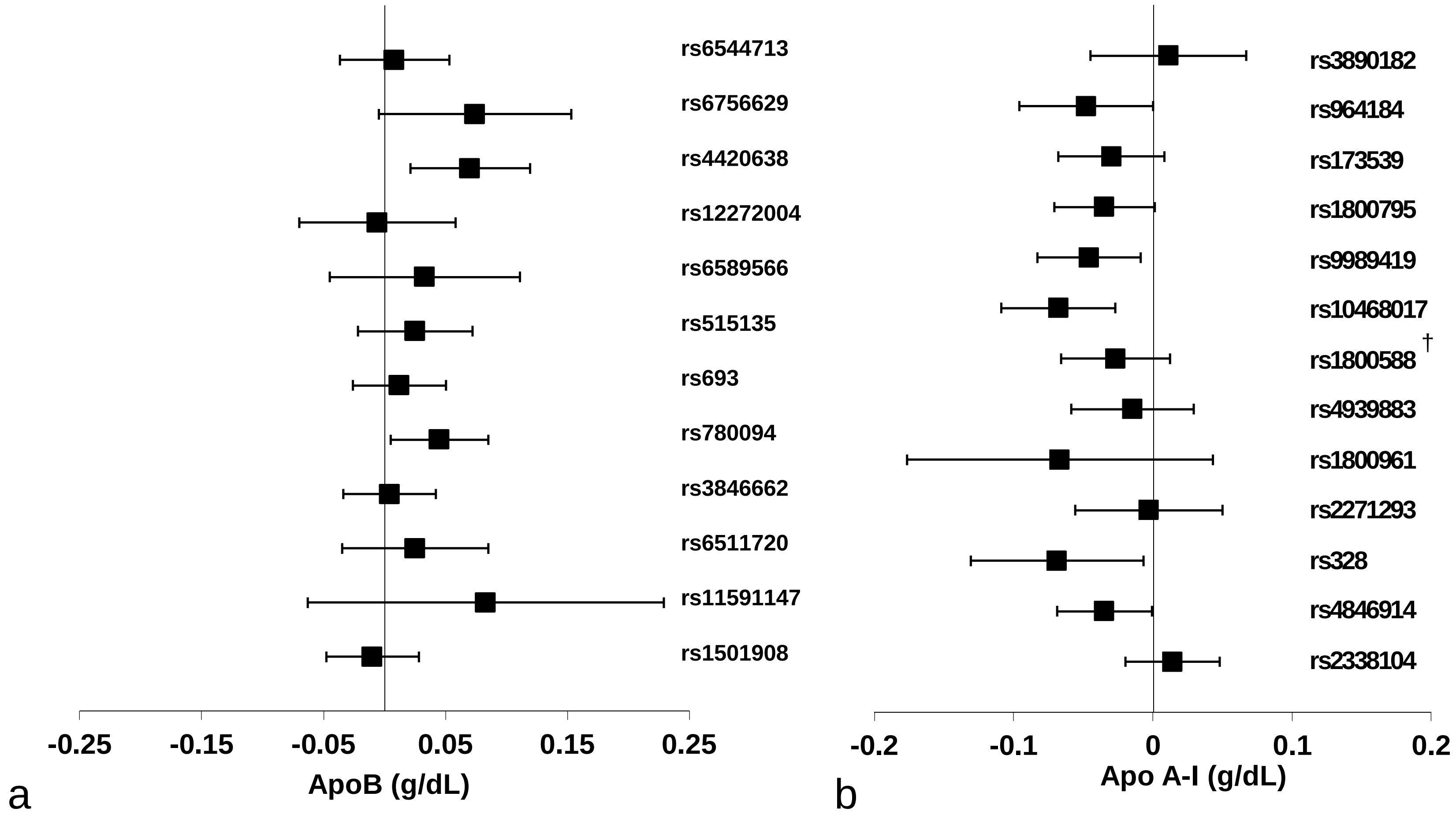

Supplementary [illegible]igure 2
The effect of LDL-C-associated SNPs on apo B (a) and HDL-C-associated SNPs on apo A-I (b) at baseline.
Data are presented as the effect size ± 95% CI from the meta-analysis of summary statistics from linear regression analyses performed in the three ethnicities between (a) individual LDL-C-SNPs and apo B and (b) individual HDL-C SNPs and apo A-I at baseline. The models were adjusted for age, gender and BMI.
† rs1800588 (LIPC) was significantly heterogeneous ($P<0.0001$) for the 3 ethnicities, the meta-analysed data for the White and Asian subgroups only (Heterogeneity = 0.256) are presented for this SNP.
