# Supplementary material for: Genetic predisposition influences plasma lipids of participants on habitual diet, but not the response to reductions in dietary intake of saturated fatty acids
Source: Atherosclerosis. 2011 Apr;215(2):421–7. doi: 10.1016/j.atherosclerosis.2010.12.039 (PMC3407860; doi:10.1016/j.atherosclerosis.2010.12.039)
Supplement: Supplementary file 3 [file mmc3.doc]

Supplementary Table 1. SNPs identified as risk alleles in previous GWAS studies, the trait for which they are associated and the frequency in this population

| Marker | Gene  (nearby gene) | SNP type | Risk allele^1^ | Risk allele Freq.^2^  (White) | Risk allele Freq.^2^  (Black African) | Risk allele Freq.^2^  (S, SE Asian) | Associated trait^3^ |
| --- | --- | --- | --- | --- | --- | --- | --- |
|  |  |  |  |  |  |  |  |
| rs3890182 | ABCA1 | Intronic | A | 0.13 | 0.12 | 0.05* | HDLC |
| rs964184 | APOA1-C3-A4-A5 | Intergenic | G | 0.14 | 0.24* | 0.25* | HDLC |
| rs173539 | CETP | Intergenic | C | 0.67 | 0.74 | 0.64 | HDLC |
| rs1800775 | CETP | Intergenic | C | 0.41 | 0.02* | 0.24 | HDLC |
| rs9989419 | CETP | Intergenic | A | 0.38 | 0.67* | 0.39 | HDLC |
| rs10468017 | LIPC | Intergenic | C | 0.85 | 0.80 | 0.71* | HDLC |
| rs1800588 | LIPC | Intergenic | C | 0.76 | 0.51* | 0.83 | HDLC |
| rs4939883 ^3^ | LIPG | Intergenic | T  C | 0.85 | 0.5* | 0.79 | HDLC  TC |
| rs1800961 | HNF4A | Coding | T | 0.03 | 0.00 | 0.01 | HDLC |
| rs2271293 | LCAT | Intergenic | A^4^ | 0.88 | 0.95* | 0.74* | HDLC |
| rs328 | LPL | Coding | C | 0.90 | 0.94 | 0.98* | HDLC  TG |
| rs4846914 | GALNT2 | Intronic | G | 0.40 | 0.90* | 0.57* | HDLC  TG |
| rs2338104 | MMAB,MVK | Intergenic | C | 0.47 | 0.23* | 0.61* | HDLC |
| rs6544713 | ABCG8 | Intergenic | T | 0.3 | 0.15* | 0.3 | LDLC |
| rs6756629 | ABCG5 | Coding | G | 0.93 | 0.93 | 0.99* | LDLC  TC |
| rs4420638 | APOE – C1-C4-C2 | Intergenic | G | 0.18 | 0.14 | 0.14 | LDLC |
| rs12272004 | APO(A1/A4A5/C3) | Intergenic | A | 0.09 | 0.17* | 0.08 | LDLC  TC  TG |
| rs6589566 | APOA5 | Intergenic | G | 0.07 | 0.01 | 0.18* | LDLC |
| rs515135 | APOB | Intergenic | C | 0.81 | 0.57* | 0.89* | LDLC |
| rs693 | APOB | Coding | T | 0.49 | 0.73* | 0.67* | LDLC  TC  TG |
| rs780094 | GCKR | Intronic | A | 0.39 | 0.16* | 0.25* | LDLC |
| rs3846662 | HMGCR | Intronic | C | 0.38 | 0.88* | 0.56* | LDLC  TC |
| rs6511720 | LDLR | Intronic | G^5^ | 0.88 | 0.89 | 0.90 | LDLC |
| rs11591147 | PCSK9 | Coding | G | 0.98 | 1.0 | 0.99 | LDLC |
| rs1501908 | TIMD4, HAVCR1 | Intergenic | C | 0.37 | 0.71* | 0.34 | LDLC |
| rs28927680 | APOA1-C3-A4-A5, ZNF259, BUD13 | Intergenic | G | 0.91 | 0.83* | 0.92 | TG |
| rs7557067 | APOB | Intergenic | G | 0.24 | 0.16 | 0.48* | TG |
| rs1260326 | GCKR | Coding | T | 0.4 | 0.14* | 0.23* | TG |
| rs2954029 | TRIB1 | Intergenic | A | 0.5 | 0.61* | 0.71* | TG |
| rs17145738 | BCL7B, TBL2, MLXIPL | Intergenic | C | 0.88 | 0.89 | 0.88 | TG |

* P<0.05 risk allele frequency in Black African S, SE Asian *cf* White

1. Published from GWAS findings in White population studies (9-16)

2. Allele frequency of the denoted risk allele in this population stratified by self-reported ethnicity

3. rs4939883 risk allele was assigned as T for HDLC T risk allele with HDLC (9, 12) and G (C) for TC (12)

4. rs2271293 risk allele denoted as G from (1) although A was found to be the risk allele in (12)

5. rs6511720 G allele associated with higher LDLC in (3, 5) but lower LDLC in (12)
