# Supplementary material for: Genetic predisposition influences plasma lipids of participants on habitual diet, but not the response to reductions in dietary intake of saturated fatty acids
Source: Atherosclerosis. 2011 Apr;215(2):421–7. doi: 10.1016/j.atherosclerosis.2010.12.039 (PMC3407860; doi:10.1016/j.atherosclerosis.2010.12.039)
Supplement: Supplementary file 4 [file mmc4.pdf]

Supplementary Table 2

Association between individual risk SNPs and total, LDL and HDL cholesterol, triglyceride[illegible]o A-I and apo B at baseline

		White				S, SE Asian				Black-African				Meta-analysis				
	Gene	Effect	SE	P	n	Effect	SE	P	n	Effect	SE	P	n	Heterogeneity	Effect	CI	CI	P
Association of TC SNPs withTC at baseline																		
rs4939883	LIPG	0.12	0.08	0.15	393	0.15	0.24	0.55	46	0.13	0.23	0.56	39	0.99	0.12	-0.02	0.27	0.09
rs6756629	ABCG5	0.40	0.13	0.002	393	0.04	0.50	0.93	46	0.18	0.53	0.74	39	0.73	0.37	0.13	0.60	0.003
rs12272004	APO(A1/A4A5/C3)	0.08	0.11	0.48	393	0.24	0.26	0.36	46	-0.29	0.37	0.43	39	0.50	0.08	-0.12	0.27	0.45
rs693	APOB	0.09	0.07	0.18	392	0.22	0.21	0.30	46	0.03	0.21	0.89	39	0.79	0.10	-0.02	0.21	0.12
rs3846662	HMGCR	-0.04	0.07	0.58	381	-0.11	0.23	0.62	41	-0.20	0.20	0.33	37	0.72	-0.06	-0.18	0.06	0.35
Association of LDL-C SNPs with LDL-C at baseline																		
rs6544713	ABCG8	-0.06	0.06	0.38	392	0.19	0.21	0.38	46	-0.04	0.25	0.86	39	0.55	-0.04	-0.15	0.08	0.54
rs6756629	ABCG5	0.33	0.11	0.003	391	-0.03	0.44	0.94	46	0.23	0.47	0.62	39	0.71	0.31	0.10	0.51	0.003
rs4420638	APOE	0.22	0.07	0.003	384	0.15	0.19	0.45	46	0.06	0.30	0.84	39	0.82	0.21	0.07	0.34	0.002
rs12272004	APO(A1/A4A5/C3)	0.05	0.09	0.57	391	0.08	0.23	0.74	46	-0.34	0.33	0.30	39	0.50	0.03	-0.14	0.20	0.72
rs6589566	APOA5	-0.08	0.12	0.47	384	0.26	0.30	0.39	46	-0.26	0.41	0.53	39	0.50	-0.05	-0.26	0.15	0.61
rs515135	APOB	0.10	0.07	0.16	390	-0.20	0.22	0.37	45	0.42	0.25	0.10	39	0.18	0.09	-0.03	0.22	0.14
rs693	APOB	0.05	0.06	0.34	390	0.13	0.19	0.48	46	0.10	0.19	0.61	39	0.91	0.06	-0.04	0.16	0.23
rs780094	GCKR	0.08	0.06	0.19	391	0.29	0.18	0.11	46	0.10	0.23	0.68	37	0.53	0.10	-0.01	0.20	0.07
rs3846662	HMGCR	-0.01	0.06	0.90	379	-0.13	0.20	0.51	41	-0.04	0.18	0.83	37	0.82	-0.02	-0.12	0.08	0.73
rs6511720	LDLR	0.08	0.09	0.35	389	0.18	0.26	0.50	46	-0.01	0.29	0.96	39	0.89	0.08	-0.07	0.24	0.30
rs11591147	PCSK9	-0.11	0.30	0.71	392	0.48	0.36	0.19	46	0.43	0.45	0.35	39	0.38	0.19	-0.21	0.60	0.35
rs1501908	TIMD4, HAVCR1	0.02	0.06	0.77	388	0.18	0.16	0.28	45	0.03	0.19	0.86	38	0.64	0.03	-0.07	0.14	0.51
Association of HDL-C SNPs with HDL-C at baseline																		
rs3890182	ABCA1	0.00	0.03	0.95	392	-0.00	0.08	0.98	45	0.08	0.11	0.46	39	0.54	0.00	-0.05	0.05	0.89
rs964184	APOA1-C3-A4-A5	-0.03	0.03	0.21	394	0.00	0.08	0.97	44	-0.11	0.09	0.21	38	0.61	-0.04	-0.08	0.01	0.13
rs173539	CETP	-0.05	0.02	0.01	388	-0.05	0.06	0.39	46	-0.13	0.07	0.05	39	0.54	-0.06	-0.10	-0.02	0.002
rs1800775	CETP	-0.02	0.02	0.32	386	-0.09	0.06	0.18	46	-0.01	0.08	0.92	39	0.58	-0.02	-0.06	0.01	0.18
rs9989419	CETP	-0.02	0.02	0.36	391	-0.10	0.06	0.10	45	-0.12	0.06	0.05	37	0.15	-0.04	-0.07	0	0.05
rs10468017	LIPC	-0.06	0.02	0.005	385	-0.06	0.07	0.39	46	-0.05	0.08	0.56	39	0.98	-0.06	-0.10	-0.02	0.003
rs1800588	LIPC	-0.09	0.02	<0.0001	392	-0.15	0.06	0.01	46	0.23	0.06	0.001	39	**<0.0001**				
rs4939883	LIPG	-0.01	0.02	0.77	393	-0.05	0.08	0.56	46	-0.10	0.07	0.16	39	0.44	-0.02	-0.06	0.02	0.38
rs1800961	HNF4A	-0.07	0.06	0.23	392	-0.16	0.16	0.34	45	0.34	0.30	0.26	38	0.345	-0.07	-0.18	0.04	0.22
rs2271293	LCAT	0.01	0.03	0.60	394	-0.04	0.08	0.64	46	-0.11	0.12	0.34	39	0.50	0.00	-0.05	0.05	0.92
rs328	LPL	-0.03	0.03	0.37	391	-0.21	0.10	0.04	43	-0.02	0.15	0.88	39	0.21	-0.05	-0.11	0.01	0.12
rs4846914	GALNT2	-0.02	0.02	0.19	390	-0.10	0.05	0.04	46	-0.03	0.06	0.60	39	0.34	-0.04	-0.07	-0.00	0.04
rs2338104	MMAB, MVK	0.01	0.02	0.69	389	0.01	0.06	0.87	45	-0.00	0.07	0.95	39	0.97	0.01	-0.03	0.04	0.69
Association of TG SNPs with lnTG at baseline																		
	APOA1-C3-A4,	-0.05	0.05	0.32	391	-0.31	0.14	0.03	46	0.23	0.15	0.14	38	**0.04**				

rs28927680	ZNF259, BUD13															
rs7557067	APOB	0.01	0.04	0.75	392	-0.03	0.09	0.77	46	-0.04	0.09	0.68	39	0.84	0.00	-0.06	0.06	0.97
rs1260326	GCKR	0.09	0.03	0.004	388	0.03	0.09	0.76	45	-0.06	0.09	0.51	38	0.25	0.07	0.02	0.13	0.01
rs2954029	TRIB1	0.01	0.03	0.79	389	0.05	0.09	0.60	46	0.03	0.08	0.72	39	0.90	0.01	-0.04	0.07	0.61
rs17145738	BCL7B, TBL2	0.16	0.05	0.002	392	0.10	0.10	0.33	45	0.26	0.11	0.02	38	0.55	0.16	0.08	0.24	<0.0001
rs328	LPL	0.04	0.05	0.50	390	0.50	0.15	0.002	43	-0.03	0.18	0.88	39	**0.01**				
rs4846914	GALNT2	-0.03	0.03	0.26	389	-0.02	0.07	0.79	46	-0.04	0.07	0.56	39	0.98	-0.03	-0.08	0.02	0.20
rs12272004	APO(A1/A4A5/C3)	0.06	0.05	0.25	392	0.04	0.11	0.72	46	-0.07	0.13	0.61	39	0.67	0.04	-0.05	0.13	0.34
rs693	APOB	0.04	0.03	0.12	391	0.03	0.09	0.74	46	0.14	0.07	0.07	39	0.51	0.06	0.01	0.11	0.03
Association of HDL-C SNPs with apo A-I at baseline																
rs3890182	ABCA1	-0.03	0.03	0.36	373	-0.03	0.06	0.65	45	0.06	0.10	0.54	37	0.79	0.01	-0.05	0.07	0.69
rs964184	APOA1-C3-A4-A5	0.00	0.03	0.85	375	-0.06	0.06	0.35	44	0.09	0.08	0.28	36	0.42	-0.05	-0.10	0.001	0.65
rs173539	CETP	-0.03	0.02	0.12	370	0.00	0.05	0.96	46	-0.02	0.06	0.70	37	0.72	-0.03	-0.06	0.01	0.13
rs1800775	CETP	-0.03	0.02	0.11	367	-0.01	0.05	0.77	46	-0.09	0.06	0.18	37	0.59	-0.04	-0.07	0.001	0.05
rs9989419	CETP	-0.03	0.02	0.09	372	-0.07	0.05	0.10	45	-0.04	0.05	0.48	35	0.14	-0.05	-0.08	-0.01	0.01
rs10468017	LIPC	-0.05	0.02	0.02	366	-0.06	0.05	0.29	46	0.06	0.07	0.36	37	0.99	-0.07	-0.11	-0.03	0.001
rs1800588	LIPC	-0.03	0.02	0.21	373	-0.03	0.05	0.48	46	0.13	0.06	0.04	37	**<0.0001**				
rs4939883	LIPG	-0.01	0.02	0.78	374	0.00	0.06	0.95	46	-0.11	0.06	0.07	37	0.38	-0.02	-0.06	0.03	0.49
rs1800961	HNF4A	-0.03	0.06	0.65	373	-0.16	0.12	0.18	45	0.06	0.26	0.81	36	0.25	-0.07	-0.18	0.04	0.24
rs2271293	LCAT	-0.02	0.03	0.41	375	-0.08	0.06	0.16	46	-0.06	0.10	0.57	37	0.76	-0.00	-0.06	0.05	0.89
rs328	LPL	-0.05	0.03	0.10	372	0.02	0.08	0.81	43	-0.05	0.13	0.73	37	0.36	-0.07	-0.13	-0.01	0.03
rs4846914	GALNT2	0.00	0.02	0.80	372	-0.07	0.04	0.10	46	-0.12	0.05	0.01	37	0.36	-0.04	-0.07	0.00	0.04
rs2338104	MMAB, MVK	-0.01	0.02	0.59	370	-0.02	0.04	0.63	45	0.08	0.06	0.22	37	0.93	0.01	-0.02	0.05	0.43
Association of LDL-C SNPs with apo B at baseline																
rs6544713	ABCG8	0.01	0.02	0.84	375	0.06	0.08	0.43	46	-0.01	0.07	0.92	37	0.77	0.01	-0.04	0.05	0.71
rs6756629	ABCG5	0.08	0.04	0.08	374	0.07	0.16	0.64	46	0.05	0.14	0.73	37	0.98	0.07	-0.01	0.15	0.07
rs4420638	APOE	0.07	0.03	**0.01**	368	0.11	0.07	0.13	46	0.02	0.09	0.82	37	0.73	0.07	0.02	0.12	0.006
rs12272004	APO(A1/A4A5/C3)	-0.01	0.04	0.80	374	0.04	0.08	0.66	46	-0.04	0.10	0.66	37	0.82	-0.01	-0.07	0.06	0.85
rs6589566	APOA5	0.06	0.05	0.22	367	-0.06	0.11	0.58	46	-0.01	0.12	0.94	37	0.58	0.03	-0.05	0.11	0.40
rs515135	APOB	0.01	0.03	0.64	373	0.03	0.08	0.69	45	0.12	0.08	0.13	37	0.42	0.03	-0.02	0.07	0.30
rs693	APOB	0.00	0.02	0.91	374	0.07	0.07	0.28	46	0.04	0.06	0.49	37	0.54	0.01	-0.03	0.05	0.52
rs780094	GCKR	0.05	0.02	**0.02**	374	0.03	0.07	0.62	46	0.01	0.07	0.94	35	0.81	0.05	0.01	0.09	0.02
rs3846662	HMGCR	0.03	0.02	0.21	363	-0.09	0.07	0.19	41	-0.08	0.05	0.16	35	0.07	0.00	-0.03	0.04	0.83
rs6511720	LDLR	0.02	0.03	0.53	373	0.09	0.09	0.34	46	-0.02	0.10	0.86	37	0.72	0.03	-0.04	0.08	0.42
rs11591147	PCSK9	-0.04	0.11	0.73	375	0.21	0.13	0.10	46	0.11	0.15	0.45	37	0.33	0.08	-0.06	0.23	0.27
rs1501908	TIMD4, HAVCR1	-0.01	0.02	0.61	371	-0.03	0.06	0.62	45	0.01	0.05	0.80	36	0.68	-0.01	-0.04	0.03	0.61

Data are the co-efficents of associations of SNP with the trait at baseline presented as effect size, standard error and P derived from linear regression models. The linear regression models were of SNP against trait at baseline adjusted for age, gender and BMI. Data are presented for each ethnic sub-group. The summary statistics were meta-analysed and the effect size (95% CI), P and heterogeneity are shown, unless there was significant heterogeneity between ethnicities (P<0.05 for the test of heterogeneity). Triglyceride data was logged for analysis and is presented in that form. The number of participants (n) in each subgroup is indicated.
