# Supplementary material for: Genetic predisposition influences plasma lipids of participants on habitual diet, but not the response to reductions in dietary intake of saturated fatty acids
Source: Atherosclerosis. 2011 Apr;215(2):421–7. doi: 10.1016/j.atherosclerosis.2010.12.039 (PMC3407860; doi:10.1016/j.atherosclerosis.2010.12.039)
Supplement: Supplementary file 5 [file mmc5.pdf]

Supplementary Table 3

Association between individual risk SNPs and the change in total, LDL and HDL cholesterol, triglycerides, apo A-I and apo B in response to 24 weeks dietary intervention on a reference, low fat or high mono-unsaturated fat diet

		White				S, SE Asian				Black-African				Meta-analysis				
	Gene	Effect	SE	P	n	Effect	SE	P	n	Effect	SE	P	n	Heterogeneity	Effect	CI	CI	P
Association of TC SNPs with change in TC																		
rs4939883	LIPG	0.01	0.05	0.89	369	-0.02	0.14	0.86	46	0.02	0.14	0.86	38	0.96	0.01	-0.08	0.09	0.90
rs6756629	ABCG5	-0.08	0.08	0.31	369	0.33	0.27	0.24	46	0.29	0.31	0.36	38	0.20	-0.03	-0.18	0.12	0.69
rs12272004	APO(A1/A4A5/C3)	-0.04	0.07	0.54	369	0.02	0.15	0.91	46	-0.01	0.22	0.98	38	0.93	-0.03	-0.15	0.09	0.62
rs693	APOB	-0.03	0.04	0.43	369	-0.19	0.12	0.13	46	-0.26	0.11	0.03	38	0.09	-0.07	-0.14	0.00	0.06
rs3846662	HMGCR	-0.01	0.04	0.75	358	0.16	0.11	0.17	41	-0.31	0.15	0.04	36	**0.04**				
Association of LDL-C SNPs with change in LDL-C																		
rs6544713	ABCG8	-0.02	0.04	0.69	368	0.13	0.12	0.30	46	0.07	0.15	0.62	38	0.46	0.00	-0.07	0.07	0.95
rs6756629	ABCG5	-0.04	0.07	0.60	367	0.29	0.23	0.22	46	0.09	0.28	0.76	38	0.38	-0.01	-0.13	0.12	0.94
rs4420638	APOE	-0.03	0.05	0.54	361	0.01	0.11	0.90	46	-0.03	0.18	0.88	38	0.94	-0.02	-0.10	0.06	0.59
rs12272004	APO(A1/A4A5/C3)	0.01	0.06	0.89	367	0.01	0.13	0.91	46	0.11	0.19	0.59	38	0.89	0.02	-0.08	0.12	0.76
rs6589566	APOA5	-0.05	0.07	0.45	360	0.19	0.17	0.25	46	0.30	0.24	0.21	38	0.17	0.01	-0.12	0.13	0.93
rs515135	APOB	0.00	0.04	0.90	366	0.12	0.13	0.34	45	-0.05	0.16	0.75	38	0.62	0.01	-0.06	0.09	0.75
rs693	APOB	-0.01	0.03	0.83	367	-0.13	0.10	0.22	46	-0.20	0.10	0.07	38	0.14	-0.03	-0.09	0.03	0.27
rs780094	GCKR	-0.03	0.03	0.42	367	-0.12	0.10	0.24	46	0.16	0.13	0.23	36	0.23	-0.03	-0.09	0.04	0.42
rs3846662	HMGCR	0.02	0.03	0.59	356	0.09	0.09	0.33	41	-0.14	0.13	0.32	36	0.38	0.02	-0.04	0.08	0.55
rs6511720	LDLR	0.05	0.05	0.37	366	-0.23	0.14	0.11	46	0.28	0.16	0.10	38	**0.05**				
rs11591147	PCSK9	0.13	0.17	0.45	368	0.34	0.19	0.09	46	0.37	0.31	0.24	38	0.66	0.25	0.01	0.48	0.04
rs1501908	TIMD4, HAVCR1	-0.05	0.03	0.14	364	0.04	0.10	0.69	45	-0.11	0.12	0.37	37	0.58	-0.04	-0.10	0.02	0.15
Association of HDL-C SNPs with change in HDL-C																		
rs3890182	ABCA1	-0.02	0.02	0.29	368	-0.02	0.06	0.75	45	0.02	0.05	0.72	38	0.76	-0.02	-0.05	0.02	0.36
rs964184	APOA1-C3-A4-A5	-0.02	0.02	0.17	370	0.02	0.05	0.65	44	-0.04	0.04	0.39	37	0.64	-0.02	-0.05	0.01	0.16
rs173539	CETP	0.00	0.01	0.87	365	-0.02	0.04	0.56	46	0.02	0.03	0.49	38	0.65	-0.00	-0.02	0.02	0.95
rs1800775	CETP	0.00	0.01	0.72	363	-0.01	0.04	0.81	46	-0.03	0.03	0.46	38	0.84	-0.01	-0.03	0.02	0.52
rs9989419	CETP	0.00	0.01	0.97	367	0.04	0.04	0.35	45	0.02	0.03	0.57	36	0.60	0.01	-0.02	0.03	0.64
rs10468017	LIPC	0.00	0.01	0.92	361	-0.08	0.04	0.06	46	-0.07	0.04	0.10	38	0.07	-0.02	-0.04	0.01	0.19
rs1800588	LIPC	-0.03	0.01	0.08	368	0.04	0.04	0.37	46	0.01	0.04	0.76	38	0.26	-0.02	-0.04	0.01	0.25
rs4939883	LIPG	0.00	0.02	0.98	369	-0.03	0.05	0.47	46	-0.03	0.03	0.40	38	0.64	-0.01	-0.03	0.02	0.55
rs1800961	HNF4A	-0.04	0.04	0.31	368	0.04	0.11	0.73	45	0.08	0.14	0.55	37	0.58	-0.02	-0.09	0.05	0.52
rs2271293	LCAT	0.00	0.02	0.94	370	0.02	0.05	0.71	46	-0.04	0.05	0.42	38	0.67	-0.00	-0.03	0.03	0.95
rs328	LPL	-0.01	0.02	0.54	367	-0.03	0.08	0.68	43	0.09	0.06	0.16	38	0.27	-0.00	-0.04	0.03	0.84
rs4846914	GALNT2	0.03	0.01	**0.03**	367	0.01	0.03	0.77	46	-0.02	0.03	0.35	38	0.19	0.02	-0.00	0.04	0.11
rs2338104	MMAB, MVK	-0.02	0.01	0.18	365	-0.02	0.04	0.51	45	0.01	0.03	0.74	38	0.69	-0.01	-0.03	0.01	0.20

Association of TG SNPs with change in TG																
rs28927680	APOA1-C3-A4, ZNF259, BUD13	0.05	0.06	0.40	368	-0.05	0.15	0.76	46	0.19	0.20	0.35	37	0.64	0.05	-0.06	0.16	0.37
rs7557067	APOB	0.00	0.04	0.94	368	-0.05	0.09	0.58	46	0.07	0.11	0.54	38	0.71	0.00	-0.07	0.07	0.93
rs1260326	GCKR	0.05	0.04	0.15	364	0.01	0.09	0.95	45	0.04	0.12	0.74	37	0.88	0.05	-0.02	0.11	0.16
rs2954029	TRIB1	-0.01	0.04	0.79	365	0.09	0.09	0.31	46	-0.04	0.11	0.71	38	0.53	0.00	-0.06	0.06	0.98
rs17145738	BCL7B, TBL2	-0.02	0.06	0.76	368	-0.03	0.11	0.80	45	0.12	0.17	0.49	37	0.74	-0.01	-0.10	0.09	0.86
rs328	LPL	-0.04	0.06	0.54	366	0.09	0.19	0.64	43	-0.01	0.23	0.96	38	0.82	-0.03	-0.13	0.08	0.65
rs4846914	GALNT2	0.00	0.03	0.93	366	-0.04	0.07	0.57	46	0.02	0.09	0.83	38	0.85	-0.01	-0.07	0.05	0.80
rs12272004	APO(A1/A4A5/C3)	-0.04	0.06	0.48	368	0.06	0.11	0.58	46	-0.28	0.16	0.09	38	0.21	-0.05	-0.14	0.05	0.37
rs693	APOB	0.00	0.03	0.93	368	-0.14	0.09	0.13	46	-0.18	0.09	0.07	38	0.11	-0.04	-0.10	0.02	0.23
Association of HDL-C SNPs with change in apo A-I																
rs3890182	ABCA1	-0.01	0.02	0.46	373	-0.01	0.05	0.88	45	-0.14	0.07	0.04	37	0.19	-0.02	-0.05	0.01	0.22
rs964184	APOA1-C3-A4-A5	-0.03	0.02	0.05	375	0.06	0.04	0.12	44	0.02	0.07	0.82	36	0.08	-0.02	-0.05	0.01	0.29
rs173539	CETP	-0.02	0.01	0.11	370	-0.03	0.03	0.34	46	0.05	0.05	0.32	37	0.34	-0.02	-0.04	0.01	0.13
rs1800775	CETP	-0.01	0.01	0.25	367	0.04	0.03	0.20	46	-0.07	0.05	0.19	37	0.14	-0.01	-0.03	0.01	0.35
rs9989419	CETP	-0.01	0.01	0.29	372	0.03	0.03	0.31	45	0.04	0.05	0.44	35	0.26	0.00	-0.03	0.02	0.70
rs10468017	LIPC	0.01	0.01	0.70	366	-0.03	0.04	0.38	46	-0.09	0.05	0.10	37	0.15	0.00	-0.03	0.02	0.73
rs1800588	LIPC	-0.02	0.01	0.19	373	-0.03	0.03	0.36	46	0.05	0.05	0.34	37	0.41	-0.02	-0.04	0.01	0.19
rs4939883	LIPG	0.01	0.01	0.71	374	0.04	0.04	0.32	46	0.10	0.05	0.05	37	0.15	0.02	-0.01	0.04	0.21
rs1800961	HNF4A	-0.04	0.04	0.31	373	0.03	0.09	0.75	46	-0.06	0.21	0.80	36	0.78	-0.03	-0.09	0.04	0.40
rs2271293	LCAT	0.00	0.02	0.79	375	0.08	0.04	0.05	46	-0.13	0.08	0.09	37	**0.03**				
rs328	LPL	0.00	0.02	0.83	372	-0.07	0.06	0.23	45	-0.07	0.10	0.49	37	0.47	-0.01	-0.05	0.02	0.48
rs4846914	GALNT2	-0.01	0.01	0.59	372	0.03	0.03	0.25	46	-0.01	0.05	0.90	37	0.44	0.00	-0.02	0.02	0.95
rs2338104	MMAB, MVK	-0.01	0.01	0.47	370	-0.03	0.03	0.26	45	-0.01	0.05	0.90	37	0.73	-0.01	-0.03	0.01	0.27
Association of LDL-C SNPs with change in apo B																
rs6544713	ABCG8	-0.01	0.01	0.62	375	-0.05	0.05	0.30	46	0.10	0.05	0.04	37	**0.05**				
rs6756629	ABCG5	-0.01	0.03	0.74	374	-0.06	0.09	0.54	46	0.13	0.09	0.15	37	0.25	0.00	-0.05	0.05	0.95
rs4420638	APOE	0.00	0.02	0.83	368	-0.05	0.05	0.33	46	0.07	0.06	0.22	37	0.28	0.00	-0.03	0.03	0.85
rs12272004	APO(A1/A4A5/C3)	-0.03	0.02	0.14	374	0.07	0.05	0.14	46	0.08	0.06	0.21	37	**0.05**				
rs6589566	APOA5	0.00	0.03	0.86	367	0.05	0.07	0.48	46	-0.02	0.08	0.77	37	0.72	0.00	-0.05	0.05	0.99
rs515135	APOB	0.01	0.02	0.53	373	-0.02	0.05	0.70	45	0.00	0.06	0.96	37	0.84	0.01	-0.02	0.03	0.64
rs693	APOB	0.00	0.01	0.92	374	-0.03	0.04	0.44	46	-0.06	0.04	0.13	37	0.29	-0.01	-0.03	0.01	0.44
rs780094	GCKR	0.00	0.01	0.89	374	-0.02	0.04	0.61	46	0.09	0.04	0.04	35	0.09	0.00	-0.02	0.03	0.73
rs3846662	HMGCR	0.00	0.01	0.78	363	0.02	0.05	0.73	41	0.04	0.05	0.47	35	0.69	0.00	-0.02	0.02	0.99
rs6511720	LDLR	0.00	0.02	0.88	373	0.08	0.06	0.16	46	-0.12	0.07	0.08	37	0.07	0.00	-0.03	0.04	0.87
rs11591147	PCSK9	0.08	0.07	0.21	375	0.03	0.08	0.76	46	-0.03	0.10	0.80	37	0.64	0.04	-0.05	0.13	0.36
rs1501908	TIMD4, HAVCR1	-0.02	0.01	0.10	371	0.02	0.04	0.58	45	-0.04	0.04	0.31	36	0.47	-0.02	-0.04	0.00	0.10

Data are the co-efficients of associations of SNP with the change in trait (final value-baseline value) presented as effect size, standard error and P derived from linear regression models. The linear regression models were of SNP against change in trait adjusted for age, gender BMI and MUFA or LF diet. Data are presented for each ethnic sub-group. The summary statistics were meta-analysed and the effect size (95% CI), P and heterogeneity are shown, unless there

was significant heterogeneity between ethnicities ($P<0.05$ for the test of heterogeneity). Negative values represent an association with a reduction in trait following 24 weeks intervention. The number of participants (n) in each subgroup is indicated.
